# The Effect of Life Coaching on Post‐Traumatic Growth and Well‐Being in Breast Cancer Survivors: A Randomized Controlled Trial

**DOI:** 10.1002/pon.70295

**Published:** 2025-10-09

**Authors:** Wing Lam Tock, Christine Maheu, Sophie Blondin, Virginia Lee, Sabine Neuman, Sarkis Meterissian

**Affiliations:** ^1^ Centre de recherche du Centre hospitalier de l’Université de Montréal Montréal QC Canada; ^2^ Department of Social and Preventive Medicine École de santé publique Université de Montréal Montréal QC Canada; ^3^ Ingram School of Nursing Faculty of Medicine and Health Sciences McGill University & Research Institute, McGill University Health Centre Montréal QC Canada; ^4^ Clinique Méno‐Joie Montréal QC Canada; ^5^ Supportive & Palliative Care Programs and Services Department of Nursing McGill University Health Centre Montréal QC Canada; ^6^ Université Paris 1 Panthéon‐Sorbonne Paris France; ^7^ Department of Surgery McGill University Health Centre Montréal QC Canada; ^8^ Department of Oncology McGill University Health Centre Montréal QC Canada

**Keywords:** breast cancer survivors, cancer, fear of cancer recurrence, life coach intervention, oncology, post‐traumatic growth, psycho‐oncology, quality of life, randomized controlled trial

## Abstract

**Background:**

Breast cancer survivors often experience psychosocial challenges, including fear of cancer recurrence, diminished quality of life, and are less aware of the potential for post‐traumatic growth. Life coaching is a strengths‐based, goal‐directed approach that may promote adjustment and recovery after cancer.

**Aim:**

This randomized controlled trial evaluated the effects of a life coaching intervention on post‐traumatic growth (PTG), fear of cancer recurrence (FCR), and quality of life (QoL) in breast cancer survivors post‐treatment.

**Methods:**

In a three‐arm randomized controlled trial at a single site, 90 women with stage I–III breast cancer were randomized to three study arms: (1) group + individual coaching, (2) group coaching only, or (3) control group receiving routine care. Coaching was delivered virtually over 15–18 weeks. The three study outcomes (PTG, FCR, QoL) were assessed at baseline, post‐group coaching (T1), end of intervention (T2), and 3‐month follow‐up (T3). Analyses included generalized estimating equations and analysis of covariance models.

**Results:**

Across the three study outcomes, no sustained or broad statistically significant improvements were observed for either experimental arms compared with control. At T2, arm 1 participants reported higher scores on the post‐traumatic growth inventory “new possibilities” subscale compared with control (OR = 1.078, 95% CI [1.00, 1.16], *p* = 0.0382), and arm 2 participants reported lower FCR scores than control (*p* = 0.0159); however, neither effect was maintained at T3. Significant differences were also observed for three QoL domains (ability to participate in social roles, fatigue, and pain interference). No significant differences were found for the remaining post‐traumatic growth inventory subscales or QoL domains.

**Conclusions:**

This first randomized controlled trial of life coaching in cancer survivorship found no sustained improvements in post‐traumatic growth, fear of cancer recurrence, or quality of life. Limited short‐term benefits suggest the intervention, in its current form, is unlikely to yield lasting psychosocial gains, underscoring the need to refine both content and outcome measures in future research.

**Trial registration:**

Clinical Trial Registry (NCT05020561).

## 1 Background

Advancements in cancer screening and treatment have significantly improved survival rates for breast cancer, which remains the most common cancer among women in Canada [1]. Since the early 1990s, the 5‐year survival rate has seen notable progress, leading to a growing population of breast cancer (BC) survivors [2]. While these medical advancements are encouraging, the transition to life after cancer (i.e., survivorship phase), presents its own set of psychological challenges. Adjusting to life after treatment often brings new uncertainties, such as fear of cancer recurrence (FCR) [3] and declines in quality of life (QoL) [4]. Accordingly, FCR and QoL are consistently identified in survivorship research as core outcomes that reflect both the psychological burden of cancer and the ability to resume everyday functioning [5].

Further, a cancer diagnosis can also trigger trauma responses, including post‐traumatic stress [6]. In this context, post‐traumatic growth (PTG) has gained prominence in cancer survivorship research [7]. PTG refers to positive psychological changes following trauma, such as a cancer diagnosis, which can lead to greater personal strength, improved relationships, and a renewed appreciation for life [8, 9]. Positive emotions, gratitude, and spirituality further promote PTG, emphasizing the role of emotional and spiritual well‐being [10]. Targeted health interventions such as life coaching are emerging as promising strategies to foster PTG among cancer survivors [11]. Life coaching focuses on personal goals and encompasses patient‐centered care, health literacy, self‐management support, and sustained behavior change. Key components of life coaching include assessing patient needs and resources, providing education tailored to individual concerns, facilitating access to services, empowering patients, and building self‐advocacy skills to navigate healthcare systems [12, 13]. This approach has been applied in various settings, including healthcare, to enhance psychological adaptation, self‐management, and self‐confidence [14, 15, 16].

Despite the increasing attention to PTG in BC survivors, current interventions often fall short in addressing the multifaceted challenges faced during the transition from treatment to survivorship. Existing programs frequently emphasize physical recovery or general psychological support but lack personalized strategies to foster self‐efficacy, autonomy, and sustained psychological adaptation and PTG [12, 13, 17]. Furthermore, there is limited empirical evidence specifically evaluating life coaching tailored to BC survivors, particularly in its capacity to mitigate FCR. This gap underscores the need for innovative, survivor‐centered approaches that address not only the psychological but also the social and practical aspects of life post‐treatment.

Based on this evidence, our study focused on PTG, FCR, and QoL: three key survivorship outcomes that together capture positive adaptation, fear and uncertainty, and day‐to‐day functioning. To address the gaps in current literature, we examine the effectiveness of a life coaching intervention aimed at empowering BC survivors during their transition to survivorship. The intervention combines both groups and individual coaching sessions focusing on goal‐setting, self‐management, and emotional resilience with tailored strategies to promote PTG and QoL while reducing FCR. Delivered by certified life coaches, the program emphasizes patient‐centered care, fostering a sense of agency and competence in navigating the complexities of post‐treatment life. The primary objective of this study is to assess the efficacy of group coaching and individual coaching sessions compared to routine care on three study outcomes: PTG, FCR, and QoL. This study aims to provide evidence supporting life coaching as a viable and impactful component of BC survivorship care.

## 2 Methods

### 2.1 Trial Design

This single site study was a randomized control trial (RCT) with three parallel arms. The full detail of the methods and content of the life coaching intervention have been described in detail in the published protocol [18]. The design and reporting of this study were guided by the Consolidated Standards of Reporting Trials (CONSORT) 2025 guideline for randomized controlled trials [19] (supplemental doc 1. CONSORT checklist). This study was approved by the McGill University Health Centre research ethics board. All study participants provided written or electronic informed consent. The trial was registered with clinicaltrial.gov: NCT05020561.

### 2.2 Participants

Consecutive sampling was used to recruit study participants. Participants were initially recruited from the breast clinic of the study site. Eligibility criteria included: (1) women aged 18+ diagnosed with stage I–III breast cancer; (2) completion of surgery, chemotherapy, or radiation within the past 18 months; (3) fluency in English or French; (4) ability to provide informed consent; and (5) access to reliable internet for video‐conference coaching sessions. Exclusion criteria were: (1) recurrent or stage IV breast cancer; (2) diagnosis of a second cancer; or (3) active psychiatric conditions that could hinder study participation. Sample size calculation was described in the published research protocol [18].

### 2.3 Data Collection

At baseline (T0), a trained research assistant obtained written informed consent and collected demographic information, along with the initial administration of the study outcome measures: PTG, FCR, and QoL. Following completion of the T0 assessments, participants were randomized into one of the three study arms. The first follow‐up (T1) occurred 3 weeks post‐baseline, immediately following the completion of group coaching sessions. At this time point, participants completed the second set of outcome questionnaires. The second follow‐up (T2), conducted 18 weeks post‐baseline, followed the completion of individual coaching sessions for participants in arm one. For participants in arms two and three, T2 marked their first post‐trial follow‐up. The final follow‐up (T3) occurred 30 weeks post‐baseline, approximately 3 months after T2. At this timepoint, all participants, across all study arms, completed the final set of outcome questionnaires, identical to those administered at earlier time points. All study measures were administered electronically via Qualtrics, a secured, web‐based platform, ensuring confidentiality and data integrity.

### 2.4 Trial Procedures

Recruitment for the study was conducted using both passive and active strategies. Passive recruitment included advertisements on social media platforms, such as the pages of local cancer organizations and Facebook groups, as well as the display of recruitment posters in the breast clinic waiting areas and on an application developed for patient communication at the recruiting site. These efforts allowed interested individuals to self‐refer by contacting the research team directly. Active recruitment involved members of the care team (e.g., oncology nurses and oncologists), who identified potentially eligible patients during routine clinic visits and through medical chart review. With the patient's permission, these staff members referred them to the research assistant, who explained the study, answered questions, and obtained written informed consent electronically. All participants included in this study signed the consent form and received a copy of the consent form via email.

**FIGURE 1:**
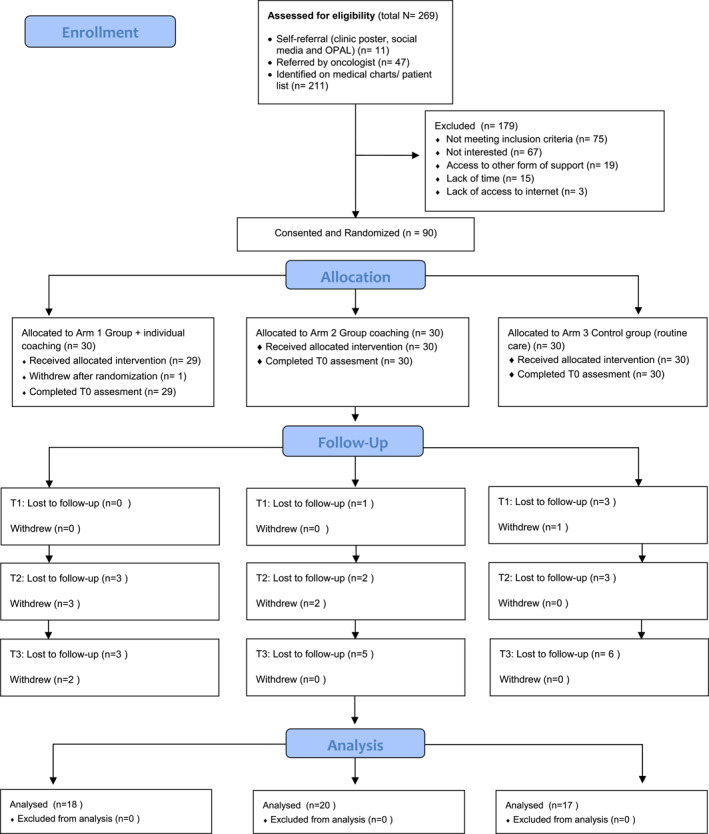
Consolidated standards of reporting trials—participants flow diagram.

Participant flow chart is shown in Figure 1. Once consent was obtained, participants were randomized into one of three study arms using a computer‐generated sequence with random blocks of six and a 1:1:1 allocation ratio. Randomization was conducted by a statistician unaffiliated with the research team to ensure blinding. For every two blocks (12 participants), the allocation was equally distributed among the three arms, and intervention groups one and two began their coaching sessions shortly thereafter.

**FIGURE 2:**
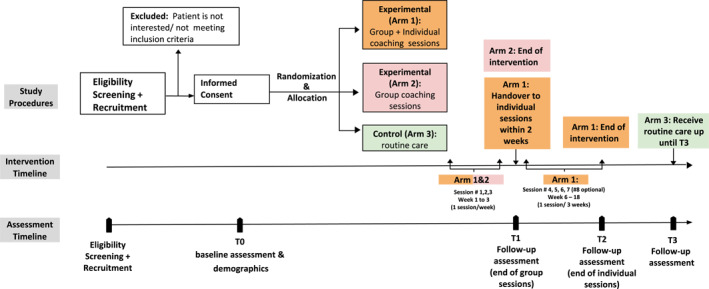
Trial procedures.

The study involved three research arms, each with distinct intervention protocols. Participants in arm one (experimental Group 1) received three group coaching sessions followed by individual coaching sessions. Participants in arm two (experimental Group 2) received only the three group coaching sessions. Participants in arm three (control group) received routine care as provided at the recruitment site. A visual representation of the trial procedures is provided in Figure 2. Detailed descriptions of what is entailed at every step of the trial process can also be found on published study protocol [18].

All life coaching sessions were conducted by professional coaches certified by the International Coaching Federation. All intervention activities were carried out virtually via video‐conferencing (e.g., Zoom). A fidelity checklist was developed to monitor the extent to which our intervention is implemented and received as intended. All life coaching sessions were recorded and reviewed for fidelity by a research team member (CM).

### 2.5 Intervention

This study's group life coaching sessions were designed to provide tools and resources to support participants in the transition from active treatment to survivorship. In our life coach intervention, Peterson and Seligman's Character Strengths and Virtues framework was used to help participants identify and apply their unique strengths toward individualized post‐treatment goals [20]. By integrating this evidence‐based strengths classification into group sessions, survivors were guided to reflect on personal resources, set meaningful objectives, and build resilience, providing a structured, strengths‐focused pathway for fostering post‐traumatic growth, enhancing quality of life, and navigating the transition to survivorship. The group coaching sessions followed a five‐step structure: identifying participant goals over a 15 week period, exploring personal strengths and experiences, reflecting on possible loss and on insights gained, linking outcomes to initial objectives, and assigning tasks to extend learning between sessions. The group format fostered a supportive environment for sharing experiences, cultivating resilience, and identifying opportunities for personal growth. A detailed description of each session, along with the materials used in the coaching intervention, is available in the online data repository for this study.

Participants in arm one proceeded to individual coaching sessions following the completion of group coaching. These individualized sessions, each lasting up to 60 min, provided a structured opportunity for participants to delve more deeply into their personal goals, address specific challenges, and articulate future aspirations. This format supported the consolidation of insights and reinforced the strengths identified during the group sessions, thereby facilitating a more tailored and transformative coaching experience.

The overall intervention emphasized the development of self‐awareness, resilience, and empowerment, equipping participants with psychological resources to navigate the post‐treatment phase. Through guided reflection, structured exercises, and peer connection, the coaching sessions offered a comprehensive framework to promote growth and enhance QoL among BC survivors.

### 2.6 Study Outcome Measures

#### 2.6.1 Demographic and Clinical Characteristics

Demographic information was collected from all participants at baseline, including age, gender, ethnicity, household income, preferred language, occupation, education level, and social support. Clinical data, including cancer stage, treatments received, and time since primary treatment completion, were extracted from participants' medical charts.

#### 2.6.2 Framework Alignment

The choice of study outcomes was guided by Peterson and Seligman's Character Strengths and Virtues framework, which emphasizes the cultivation of personal strengths, reduction of psychological barriers, and promotion of flourishing as markers of well‐being [20]. Accordingly, we selected measures that captured the activation of personal strengths (i.e., PTG), the mitigation of psychological distress (i.e., FCR), and improvements in multidimensional QoL.

#### 2.6.3 Primary Outcome: Post‐Traumatic Growth

PTG was assessed using the Posttraumatic Growth Inventory (PTGI) [21]. The PTGI is a 21‐item instrument designed to measure positive psychological changes experienced as a result of a traumatic event. Participants respond using a six‐point Likert scale. The scale comprises five subscales: *New Possibilities*, *Relating to Others*, *Personal Strength*, *Appreciation of Life*, and *Spiritual Change*. A total PTG score range from 0 to 105; higher scores indicate greater perceived growth [21]. In this study, the PTGI demonstrated acceptable internal consistency (*α* = 0.75).

#### 2.6.4 Secondary Outcome: Fear of Cancer Recurrence

FCR was measured using the six‐item Cancer Worry Scale (CWS) [22], which assesses the frequency and impact of worry about cancer recurrence. Items are rated on a four‐point Likert scale, resulting in total scores ranging from 6 to 24. Higher scores indicate greater cancer‐related worry. The internal consistency of the CWS in this study was high (*α* = 0.91). Validated cut‐off scores on the CWS‐6 indicate that totals ≥ 9 reflect high FCR, while totals ≥ 11 reflect severe FCR [22].

#### 2.6.5 Secondary Outcome: Quality of Life

QoL was assessed using the Patient‐Reported Outcomes Measurement Information System –Preference (PROPr) instrument [23, 24]. The PROPr comprises 31 items assessing eight health domains. Raw scores for each domain are converted into standardized T‐scores (mean = 50, standard deviation = 10) based on the U.S. general population norms. Interpretation depends on the domain: for symptom domain (e.g., fatigue, pain interference, anxiety, depression, sleep disturbance), higher T‐scores indicate worse symptoms; for function domains (e.g., physical function, ability to participate in social roles and activities, cognitive function), higher T‐scores indicate better functioning. In this study, internal consistency across the eight domains ranged from α = 0.82 to 0.95.

### 2.7 Data Analysis

All analyses followed an intent‐to‐treat approach, ensuring that data from all randomized participants were included regardless of session attendance or adherence to the intervention.

Descriptive statistics were computed to summarize demographic and clinical characteristics across the three study arms: arm one (experimental Group 1) group plus individual coaching; arm two (experimental Group 2) group coaching only; and arm three (control group) routine care. Continuous variables (e.g., age) were described using means and standard deviations, while categorical variables (e.g., marital status) were presented as frequencies and percentages.

Internal consistency of the outcome measures was assessed using Cronbach's alpha, calculated both across the full sample and within each study arm. All scales demonstrated acceptable to excellent internal reliability.

To evaluate overall differences between groups, a non‐parametric rank‐sum test was conducted (i.e., Kruskal–Wallis test), providing a distribution‐free comparison of scores across study arms. To assess the effect of group assignment over time, repeated measures analysis of covariance (ANCOVA) was employed, with interaction terms included to examine differential changes across timepoints. This analysis addressed (1) within‐group changes over time, (2) between‐group differences, and (3) group‐by‐time interaction effects.

Generalized Linear Models (GLM) and Generalized Estimating Equations (GEE) were used to model associations between outcome scores and group membership, with arm three (control group) serving as the reference group. These models estimated the fixed effects of the interventions while adjusting for baseline values where applicable. Assumptions regarding homogeneity of variance and group‐by‐time interactions were evaluated. Effect sizes were computed to quantify the magnitude of observed changes.

Missing outcome data, including those from participants lost to follow‐up, were addressed using multiple imputation procedures based on baseline and available follow‐up values. To assess the robustness of findings, results from the imputed datasets were compared with complete‐case analyses, and the conclusions were consistent across both approaches. All analyses were performed using SPSS version 29.0.2. Statistical significance was set at *p* < 0.05. No additional covariates were included in the models, as the randomized design was expected to minimize baseline confounding and support attribution of group differences to the intervention effects.

## 3 Results

### 3.1 Participants

A total of 269 patients were screened for eligibility from 2021 June to 2023 October. Of these, 90 individuals consented to participate and were randomized equally across the three study arms (30 participants per arm). Most participants were identified by the research assistant through medical chart review and patient lists (*n* = 211), while 47 were referred by oncologists or other healthcare professionals, and 11 participants self‐referred.

Prior to the baseline outcome assessment (T0), one participant from arm one withdrew, resulting in 89 participants completing baseline measures. Over the course of the study, an additional eight participants withdrew: five from arm one, two from arm two, and one from arm three. Furthermore, 27 participants did not complete follow‐up assessments at one or more timepoints and were considered lost to follow‐up.

By the final time point (T3), 18 participants remained in arm one, 20 in arm two, and 17 in arm three, resulting in a total of 55 participants who completed all data collection points from T0 to T3. This corresponds to an overall attrition rate of 38.9%. Although 90 participants were randomized, one participant did not complete the baseline outcome questionnaires; her demographic data were retained for transparency, but this participant was excluded from outcome analyses, resulting in a final sample size of 89 participants. The detailed flow of participants throughout the study is presented in Figure 1.

### 3.2 Demographic and Clinical Characteristics

As presented in Table 1, the final sample consisted of 90 female BC survivors, with a mean age of 50 years (SD = 6.2). The majority identified as White (80%) and Canadian‐born. More than half of participants reported being married (58%), employed (65%), and having completed a university‐level education (55%). Demographic characteristics were well‐balanced across the three study arms, supporting the assumption of comparability. On average, participants had completed primary treatment (i.e., surgery, chemotherapy, or radiation) 10.6 months (SD = 3.87) prior to study enrollment. Other clinical characteristics, including cancer stage and type of treatments received, were also comparable across groups.

**TABLE 1:** Demographic and clinical characteristics of participants at baseline.

	Experimental group 1		Experimental group 2		Control group	
Demographic characteristics	Mean	SD	Mean	SD	Mean	SD
Age (years)	49.97	10.16	56.07	9.13	51.37	7.19

	*n*	%	*N*	%	*n*	%
Gender						
Female	30	100	30	100	30	100
Ethnicity/Racial identity						
Arab	0	0	0	0	4	13.3
Black	1	3.3	0	0	0	0
Chinese	1	3.3	2	6.7	1	3.3
Latin American	1	3.3	0	0	2	6.7
South Asian	1	3.3	1	3.3	0	0
White	24	80.0	23	76.7	21	70.0
Other	2	6.7	4	13.3	2	6.7
Born in Canada						
Yes	24	80.0	21	70.0	20	66.7
No	6	20.0	9	30.0	10	33.3
Preferred language						
English	14	46.7	19	63.3	15	50.0
French	16	53.3	9	30.0	11	36.7
Other	0	0	2	6.7	4	13.3
Annual household income						
Less than $25,000	2	6.7	1	3.3	1	3.3
$25,001 ‐ $50,000	4	13.3	2	6.7	1	3.3
$50,001 ‐ $70,000	3	10.0	6	20.0	4	13.3
$75,001 ‐ $100,000	5	16.7	3	10.0	7	23.3
More than $100,001	12	40.0	13	43.3	12	40.0
Prefer not to say	4	13.3	5	16.7	5	16.7
Education						
High school or less	1	3.3	2	6.7	0	0
High school graduate	0	0	0	0	0	0
Some CEGEP	4	13.3	4	13.3	5	16.7
Some university/college	4	13.3	2	6.7	4	13.3
College/technician school degree	0	0	0	0	0	0
University degree	16	53.3	15	50.0	11	36.7
Post‐graduate degree	5	16.7	7	23.3	10	33.3
Marital status						
Married/Common law	15	50.0	18	60.0	21	70.0
Divorced/Separated	6	20.0	7	23.3	6	20.0
Single	7	23.3	3	10.0	1	3.3
Other	2	6.7	2	6.7	2	6.7
Employment/Education status						
Full‐time	12	40.0	13	43.3	9	30.0
Part‐time	1	3.3	3	10.0	6	20.0
Student	1	3.3	0	0	0	0
Unemployed: Illness/disability	8	26.7	6	20.0	8	26.7
Unemployed: Other	3	10.0	1	3.3	2	6.7
Retired	1	3.3	3	10.0	0	0
Other	4	13.3	4	13.3	5	16.7
Number of children						
0	12	40.0	8	26.7	2	6.7
1	6	20.0	5	16.7	8	26.7
2	10	33.3	11	36.7	16	53.5
3	1	3.3	5	16.7	3	10.0
4 or more	1	3.3	1	3.3	1	3.3
Number of dependent children living at home						
0	13	43.3	15	50.0	7	23.3
1	9	30.0	6	20.0	12	40.0
2	6	20.0	6	20.0	8	26.7
3	1	3.3	3	10.0	2	6.7
4 or more	1	3.3	0	0	1	3.3

Clinical characteristics						
Cancer stage						
I (*n* = 28)	6	21.4	12	42.9	10	33.3
II (*n* = 45)	17	37.8	13	28.9	15	50.0
III (*n* = 17)	7	41.2	5	29.4	5	16.7
Treatments received (responded yes)						
Chemotherapy	16	53.3	14	46.7	22	73.3
Radiation therapy	15	50.0	19	63.3	14	46.7
Surgery (lumpectomy)	3	10.0	2	6.7	2	6.7
Surgery (mastectomy)	12	40.0	9	30.0	10	33.3
Adjunctive therapy (hormonal therapy)	8	26.7	11	36.7	9	30.0
Adjunctive therapy (Immunotherapy)	6	20.0	4	13.3	7	23.3

	Mean	SD	Mean	SD	Mean	**SD**
**Time since treatment completion (in months)** ^a^	**10.25**	**3.60**	**9.52**	**3.72**	**12.03**	**3.95**

^a^ Time since treatment completion was calculated in months from the date of final primary treatment (surgery, chemotherapy, or radiation) to the baseline assessment (T0).

### 3.3 Overall Effects of the Intervention

Across the three study outcomes, GEE analyses indicated no sustained or broad between‐group differences over time. Where statistically significant differences emerged, they were generally small in magnitude, occurred at a single time point, and were not maintained at follow‐up.

### 3.4 Primary Outcome

#### 3.4.1 Post‐Traumatic Growth

Descriptive statistics for each PTGI subscale (*Relating to Others, New Possibilities, Personal Strength, Spiritual Change, and Appreciation for Life*) across all time points and study arms are presented in supplemental doc 2. Mean scores increased over time for all three groups, with the largest gains generally observed among participants in the group plus individual coaching arm, particularly for *Personal Strength* and *New Possibilities*.

GEE analyses were conducted to compare the two experimental groups to the control group on the five subscales of the PTGI. A statistically significant difference was observed for the *New Possibilities* subscale. Participants in arm one, who received group plus individual coaching sessions, demonstrated 7.8% higher odds of reporting improvement on this subscale compared to participants in arm three (control group), who received routine care (OR = 1.078, 95% CI [1.00, 1.16], *p* = 0.0382). No statistically significant differences were found for *Relating to Others*, *Spiritual Change*, or *Appreciation for Life*. See Table 2 for full GEE results across all subscales.

**TABLE 2:** Generalized estimating equation results of the three study outcomes.

	Experimental Group 2 versus. Control^a^			Experimental Group 1 versus. Control			Experimental Group 2 versus. Experimental Group 1		
Study groups	Odds ratio	95% CI	*p*‐value	Odds ratio	95% CI	*p*‐value	Odds ratio	95% CI	*p*‐value
Post‐traumatic growth									
Relating to Others	1.056	0.97–1.15	0.2194	1.071	0.98–1.17	0.1388	1.019	0.93–1.12	0.6918
New Possibilities	1.027	0.96–1.10	0.4533	1.078	1.00–1.16	0.0382*	1.055	0.98–1.14	0.1677
Personal strength	1.053	1.00–1.11	0.0594	1.048	0.99–1.10	0.0782	0.997	0.94–1.06	0.9185
Spiritual change	0.925	0.80–1.07	0.2825	0.897	0.77–1.05	0.1708	0.981	0.85–1.13	0.7940
Appreciation for life	0.997	0.89–1.12	0.9565	1.057	0.92–1.21	0.4211	1.060	0.94–1.20	0.3512
Fear of cancer recurrence									
FCR level	0.922	0.83–1.02	1.1198	0.937	0.85–1.03	0.1951	1.003	0.90–1.12	0.9655
Quality of life									
Physical function	1.021	0.96–1.08	0.4752	0.983	0.93–1.04	0.5571	0.950	0.89–1.01	0.1180
Anxiety	0.962	0.91–1.02	0.1651	0.986	0.94–1.04	0.5976	1.020	0.97–1.07	0.4329
Depression	0.998	0.95–1.04	0.9152	1.030	0.99–1.08	0.1855	1.032	0.99–1.08	0.1667
Fatigue	0.952	0.91–1.00	0.0389*	0.988	0.95–1.03	0.5779	1.043	0.99–1.10	0.0926
Sleep disturbance	0.969	0.92–1.03	0.2742	0.977	0.92–1.04	0.4550	1.011	0.96–1.07	0.7074
Social roles and activities	1.084	1.02–1.15	0.0073*	1.013	0.95–1.07	0.6777	0.27	0.87–0.98	0.0141*
Pain interference	0.937	0.89–0.99	0.0145*	0.973	0.92–1.03	0.3282	1.049	0.99–1.11	0.0872
Cognitive	1.030	0.97–1.09	0.3369	0.987	0.92–1.05	0.6980	0.951	0.89–1.01	0.1251

^a^ Note: Experimental Group 1 (*n* = 29), experimental Group 2 (*n* = 30), control group (*n* = 30).

### 3.5 Secondary Outcomes

#### 3.5.1 Fear of Cancer Recurrence

At baseline, FCR levels as measured with the CWS show scores that were in the severe range (≥11) for all three study arms (supplemental doc 2), indicating clinically meaningful levels of FCR (arm 1: 14.4 ± 5.1; arm 2: 13.9 ± 4.0; arm 3: 15.1 ± 3.3). Scores declined modestly over time in both experimental arms. Immediately after coaching completion (T2), both intervention groups reported significantly lower FCR scores than the control group (*p* = 0.0159), although these differences were not maintained at 3‐month follow‐up.

Consistent with the descriptive trends, GEE models showed no statistically significant reduction in FCR for either intervention arm compared with the control group (arm 1: OR = 0.937, 95% CI [0.85, 1.03], *p* = 0.1951; arm 2: OR = 0.922, 95% CI [0.83, 1.02], *p* = 0.1198). A direct comparison between the two intervention arms also revealed no significant difference (OR = 1.003, 95% CI [0.90, 1.12], *p* = 0.9655). While a transient improvement in FCR was observed at T2, no significant differences persisted over time.

#### 3.5.2 Quality of Life

Results from the GEE analysis showed statistically significant differences between the study arms on three of the eight QoL domains of the PROPr: *Ability to Participate in Social Roles*, *Fatigue*, and *Pain Interference*.

At baseline, there were no significant differences in scores between the three study arms for the domain *Ability to Participate in Social Roles*. While higher mean scores were observed at T2 in arm 2 compared to the other arms, overall differences across all three groups were not statistically significant at that time point. However, across all timepoints, GEE analysis indicated that participants in arm two had 8.4% greater odds of improvement in this domain compared to the control group (OR = 1.084, 95% CI [1.02, 1.15], *p* = 0.0073). Additionally, when comparing the two experimental groups directly, Arm Two showed greater improvement than arm one (OR = 0.93, 95% CI [0.87, 0.98], *p* = 0.0141).

At baseline, mean T‐scores indicated mild to moderate fatigue across all arms, with no significant differences between groups. However, across all timepoints, GEE analysis showed that participants in arm two had a 4.8% reduction in the odds of experiencing elevated fatigue compared to the control group (OR = 0.952, 95% CI [0.91, 1.00], *p* = 0.0389). However, results from ANOVA at the final follow‐up (T3) indicated that these differences were no longer statistically significant (*p* = 0.7642; rank‐sum test, *p* = 0.8832 and 0.6682), suggesting that the observed improvement in fatigue may not have been sustained over time.

In regrads to *Pain Interference*, there were no significant differences between groups at baseline. However, across all timepoints, GEE analysis indicated that participants in arm two were significantly less likely to report pain interference compared to those in the control group, with a 6.3% reduction in odds (OR = 0.937, 95% CI [0.89, 0.99], *p* = 0.0145).

## 4 Discussion

Our trial represents the first RCT evaluating the efficacy of life coaching intervention for BC survivors. Overall, the intervention did not demonstrate broad effects on PTG, FCR, or QoL. While small improvements were observed in selected domains, such as the *New Possibilities* subscale of the PTGI and transient improvements in fatigue, social role participation, and pain interference, these effects were limited in scope and not sustained at follow‐up. Taken together, the findings indicate that life coaching, as delivered in this study, did not produce consistent or durable benefits across the measured outcomes. Yet, the largely nonsignificant results reported in this study can potentially contribute to the evidence base, helping to refine outcome selection and inform the design of future life coaching interventions in the context of cancer survivorship.

The fatigue sub‐domain from the PROPr was one of the few outcomes where we observed short‐term improvements in the group coaching arm. This was clinically relevant given our recruitment criteria (≤ 18 months post‐treatment), a period when cancer‐related fatigue remains highly prevalent [25]. However, given the small magnitude of this effect, these findings should be interpreted with caution and viewed as exploratory, particularly in the context of the overall null pattern for other QoL domains.

The lack of statistically significant effects of the intervention on FCR was unexpected, particularly given the intervention's emphasis on resilience‐building. One possible explanation is that FCR may require more structured cognitive‐behavioral interventions, such as the Fear of Cancer Recurrence Therapy intervention, which specifically targets threat monitoring and intrusive worry of cancer recurrence in breast and gynecological cancer survivors [3]. Work has shown that targeted cognitive‐existential strategies, such as the “worst‐case scenario” exposure exercise, can help survivors process recurrence fears and reduce associated distress [26]. The life coaching intervention in this study did not incorporate such cognitive‐existential exposure exercises, which may have limited its ability to address entrenched cognitive patterns associated with FCR. Instead, the focus was on emotional support, values clarification, and goal setting, important elements for overall well‐being, but perhaps insufficient to directly modify the fear‐related cognitions that maintain FCR. In contrast, life coaching interventions may provide emotional support and goal‐setting strategies but may not be sufficient to disrupt cognitive patterns related to FCR. This finding highlights a gap that should be addressed in future life coaching interventions.

Our findings also highlight important considerations for interpreting the limited effects observed. Notably, our findings suggest that the participants receiving only group coaching (arm 2) showed greater improvements in selected QoL outcomes than the group receiving both group and individual coaching (arm 1). This finding suggests that the communal dynamics inherent in group settings may play a pivotal role in the recovery process for BC survivors [27], which could account for the modest short‐term changes in role functioning and social connectedness. The group setting allows participants to exchange stories and coping strategies, which can reduce isolation and promote emotional recovery [28, 29]. Adding individual sessions might have shifted focus away from the group dynamic, reducing its impact. This unexpected finding suggests that BC survivors benefit from interventions that emphasize community and shared experience, as opposed to mixed group and individual interventions. However, the benefits of group coaching were neither consistent across QoL outcomes nor sustained over time, suggesting that the intervention as delivered was not sufficient to influence broader aspects of adjustment. These results point to the value of refining both intervention content and outcome selection in order to more accurately capture the ways life coaching might contribute, if at all, to survivorship recovery.

Life coaching may be most effective when tailored to individual readiness, delivered in a format that fosters trust and engagement, and complemented by other therapeutic supports. Our findings point to the need for thoughtful integration of coaching into survivorship care, specifically targeting survivors who are seeking direction, support in goal‐setting, or connection during their transition out of treatment.

### 4.1 Limitations

One limitation of this study is the small sample size. Although we planned to recruit 120 participants based on our original power calculation [18], only 90 were enrolled due to time and funding constraints. Attrition was also higher than expected at 38.9%, which reduced our ability to detect smaller effects and conduct subgroup analyses. While the findings offer early insights, larger studies are needed to confirm the results.

In addition, our eligibility criteria did not target survivors with high psychosocial distress or poor baseline adjustment, which may have limited the magnitude of observable intervention effects. Another limitation is that although widely used and psychometrically robust, the PTGI and PROPr may not fully capture the mechanisms through which a strengths‐based, goal‐directed intervention such as life coaching might exert its effects. Life coaching aims to foster positive functioning, goal achievement, and resilience, while constructs such as empowerment, autonomy, engagement and meaning‐making were not directly assessed in this trial. The absence of measures such as the Positive emotion, Engagement, Relationships, Meaning, and Accomplishment (PERMA) Profiler, which evaluates multidimensional well‐being, or other coaching‐relevant instruments, may have limited our ability to detect subtle benefits aligned with the coaching model [30].

These limitations indicate that while this trial provides important preliminary evidence, future studies will need larger, more diverse samples, longer follow‐up, and outcomes more closely aligned with coaching processes in order to determine whether life coaching has a meaningful role in BC survivorship care.

### 4.2 Recommendations and Future Research Directions

These results underscore the importance of matching outcome measures to intervention mechanisms and considering both the intensity and timing of delivery. Future trials could incorporate well‐being instruments such as the PERMA Profiler, and measures that may more accurately evaluate coaching more accurately such as measures of resilience, autonomy, empowerment, and meaning‐making to evaluate coaching more accurately [30] In fact, to address this measurement gap within the current trial, we have already incorporated selected PERMA Profiler items into a follow‐up qualitative exit interview study with a subgroup of participants from the RCT. These in‐depth interviews will explore how survivors experienced the coaching process, including resilience‐building, empowerment, and psychological adaptation, that may not have been captured by our quantitative measures. Alongside the PERMA Profiler, other instruments such as the Psychological Well‐Being Scale [31, 32], and the Connor–Davidson Resilience Scale [33] could help future trials more precisely assess how life coaching fosters well‐being and coping capacity.

Other considerations for future trials are to extend follow‐up periods, and explore booster sessions to sustain gains. Reporting null findings, as we do here, remains essential for building an accurate evidence base and refining supportive care interventions. In addition, longitudinal studies with longer follow‐up periods are necessary to determine whether any observed benefits can be sustained beyond 3 months. Research should also focus on clarifying the mechanisms by which specific coaching techniques influence outcomes such as personal growth, coping, and QoL, and whether these effects differ depending on individual readiness or time since treatment. As those closer to treatment often report higher fatigue, pain, and FCR compared to longer‐term BC survivors, future trials should consider stratifying or adjusting for this factor to capture its potential impact.

## 5 Conclusion

In this first randomized controlled trial testing a structured life coaching program in cancer survivorship, no sustained improvements were observed on the primary outcome of post‐traumatic growth, and only limited, short‐lived effects were found on fear of recurrence and quality of life. While life coaching may offer transient benefits for some survivors, the overall pattern of results suggests that, in its current form, the intervention is unlikely to deliver broad, lasting psychosocial gains. These findings should inform refinement of both the content and delivery of life coaching interventions, as well as the selection of outcomes most aligned with their intended mechanisms of change.

## Author Contributions

Conceptualization: C.M., V.L., S.M. Methodology: C.M., V.L., S.M. Intervention fidelity and supervision: W.L.T., C.M., V.L, S.M. Formal analysis: W.L.T., C.M. Investigation: W.L.T., C.M., V.L., S.B., S.N., S.M. Resources: C.M., V.L., S.B., S.N., S.M. Data curation: W.L.T., C.M. Writing – original draft preparation: W.L.T, C.M. Writing – review and editing: W.L.T, C.M., V.L., S.B., S.N., S.M. Visualization: W.L.T., C.M. Supervision: C.M., V.L., S.M. Project administration: W.L.T., C.M., V.L., S.B., S.N., S.M. Funding acquisition: C.M., V.L., S.M. All authors have read and agreed to the published version of the manuscript.

## Ethics Statement

Research Institute of the McGill University Health Center: Research Ethics Board (REB) on 2021/07/26. This trial is registered on clinicaltirla.gov [NCT05020561].

## Conflicts of Interest

The authors declare no conflicts of interest.

## Supporting information


Supporting Information S1

**Table S1:** Descriptive statistics for study outcome by time point and study arm.

## Data Availability

Open Science Framework data repository, accessible https://osf.io/m7prd/?view_only=5c51a705cf43485aafa45c615f108ec5.
